# Research on Mental Workload of Deep-Sea Oceanauts Driving Operation Tasks from EEG Data

**DOI:** 10.3390/bioengineering10091027

**Published:** 2023-08-31

**Authors:** Xiaoguang Liu, Lu Shi, Cong Ye, Yangyang Li, Jing Wang

**Affiliations:** 1 School of Naval Architecture, Ocean & Civil Engineering, Shanghai Jiao Tong University, Shanghai 200240, China; robert954760@my.yosemite.edu; 2 Shanghai Jiao Tong University and Chiba University International Cooperative Research Center (SJTC-CU-ICRC), Shanghai 200231, China; 3 Institute of Underwater Technology, Shanghai Jiao Tong University, Shanghai 200231, Chinahector947856@my.yosemite.edu (J.W.); 4 China Ship Scientific Research Center, Wuxi 214028, China; derek954762@my.yosemite.edu

**Keywords:** oceanauts, mental workload, EEG data, multi-class support vector machine, quantum genetic algorithm

## Abstract

A person’s present mental state is closely associated with the frequency and temporal domain features of spontaneous electroencephalogram (EEG) impulses, which directly reflect neurophysiological signals of brain activity. EEG signals are employed in this study to measure the mental workload of drivers while they are operating a vehicle. A technique based on the quantum genetic algorithm (QGA) is suggested for improving the kernel function parameters of the multi-class support vector machine (MSVM). The performance of the algorithm based on the quantum genetic algorithm is found to be superior to that of other ways when other methods and the quantum genetic algorithm are evaluated for the parameter optimization of kernel function via simulation. A multi-classification support vector machine based on the quantum genetic algorithm (QGA-MSVM) is applied to identify the mental workload of oceanauts through the collection and feature extraction of EEG signals during driving simulation operation experiments in a sea basin area, a seamount area, and a hydrothermal area. Even with a limited data set, QGA-MSVM is able to accurately identify the cognitive burden experienced by ocean sailors, with an overall accuracy of 91.8%.

## 1. Introduction

Jiaolong’s deep-sea submarine voyages have been expanding, and there is a growing need for the workload evaluation of oceanauts due to China’s rapid development of deep-sea resource exploration and development. An oceanaut operates manned submarine equipment and works in a complex deep-sea environment; the main workload they bear is mental workload [1]. The classification of mental burden is one of main topics in investigations on the operating jobs of deep-sea manipulator oceanauts. In an effort to categorize and forecast levels of mental burden, current research efforts often use the subjective scale technique, primary task method, supported task method, and psychophysiological measurements [2,3,4]. The four indexes—the accurate reaction time for a primary task, the variation rate of the accurate reaction time for a subsidiary task, the weighted subjective workload rating, and the variation rate of heart rate variance—were used and tested [5] in Zhang’s two mathematical models of comprehensive mental workload indexes. Hancock et al. put predictions from a model of mental effort to the test using the Time Pools performance challenge. Data from the experiment showed that when perceived distance from a task objective rises and the effective time for action decreases, mental burden increases [6]. According to Noel et al.’s research, a tiny subset of integrated and calibrated psycho-physiological variables obtained from a single pilot on a particular day may properly categorize the mental effort for a different pilot on a different day [7]. Cantin et al. used the probing reaction time (RT) approach to quantify workload and looked at how young and older active drivers’ mental effort changed depending on how challenging the driving environment was [8]. To determine if subjective time perception might be utilized as a gauge of cognitive effort during simulated automobile driving, Baldauf et al. employed electrodermal activity and subjective assessments of mental workload (SWAT) [9].

To evaluate the mental workload related to agricultural spraying, Dey et al. compared and chose the best variants of NASA-TLX and SSWAT. The study looked at the various variants of two widely used workload rating scales in terms of their sensitivity and diagnosticity and chose the best variants of each scale for upcoming mental workload research [10]. According to Jo et al.’s proposed mathematical model in relation to the activated time of ACT-R modules, the participants’ average NASA-TLX ratings were highly correlated with the predicted values of mental workload attained using the proposed method [11]. In order to evaluate mental effort, Mouzé-Amady et al. devised a novel technique for calculating weights from qualitative fuzzy integrals and applied it to the NASA-TLX subscales of the National Aeronautics and Space Administration-Task load index [12]. To measure mental workload, Klein et al. utilized the multiple resources questionnaire (MRQ) and the Dundee stress state questionnaire (DSSQ) [13]. The results suggested a potentially applicable method to brain computer interface systems that adapt to human mental workload [14]. In a municipal traffic control center, Majid et al. employed the NASA-Task load index (TLX) to assess operators’ mental effort while observing traffic density. The findings revealed that the operators had a greater mental burden during high traffic density than during low traffic density [15]. These approaches have significant systematic mistakes, applicability restrictions, and are often contaminated by the subjective preferences of decision makers [16,17,18]. Different machine learning algorithms have been presented in this field in an effort to find an appropriate way to measure mental strain and have shown competitive performance [19,20,21]. Ke developed a comprehensive mental workload recognition model using feature selection and regression modeling. The cross-task regression performance was greatly improved [22] when the model was trained and evaluated using the most robust feature subset chosen by cross-task RFE. According to the classification findings (with the greatest five-class accurate classification rate of 88%), the location projection preservation approach can retain high enough MWL classification accuracy [23]. In order to evaluate operator workload, Grassmann et al. integrated physiological and self-report measures of mental effort. The findings indicated that including individual characteristics may minimize unexplained variance and boost the validity of workload assessments [24]. By repeatedly adding misclassified instances from the test set to the training set, Zhang et al. developed an adaptive support-vector-machine-based technique to categorize operator mental workload (MWL) into a few discrete levels to adjust the model’s performance to a new participant [25].

So et al. looked into the viability of assessing the dynamic changes in mental effort using short-term frontal EEG [26]. Despite the fact that the aforementioned studies have examined and debated the mental workload and have produced many successes, they have not yet been used to recognize the mental burden of oceanauts. In order to obtain the EEG signals of the individuals while they were driving, feature extraction and analysis were performed on deep-sea driving simulation trials. It was suggested to use QGA-MSVM to efficiently categorize the mental burden experienced by drivers.

Currently, there is not much research on measuring mental strain for oceanauts; it mostly focuses on application industries like pilots and drivers of cars. The manned submersible simulation operating system is created using 3dsMax and Unity3D. 

The remainder of this essay is structured as follows. EEG signal extraction and data processing are described in Section 2. In Section 3, a kernel function parameter optimization strategy based on a quantum evolutionary algorithm is suggested along with a mathematical explanation of data categorization. The task is designed and the platform for the simulation experiment is built in Section 4. In Section 5, the findings are examined and discussed. The key points of the text are outlined in Section 6. 

In order to develop a reasonable operation process and to take action when the mental load of the underwater crew is either too small or too large (man–machine function redistribution, the responsibility redistribution of the chief and co-pilot, and task strategy re-selection), physiological indicators are used to effectively assess the mental load of the underwater crew. 

## 2. EEG Signals and Processing

### 2.1. EEG Signals

EEG is a technique used to electrophysiologically record brain activity, including mental effort during job activities. Thirty-two Ag/AgCl scalp electrodes were placed in accordance with the worldwide 10/20 standard on a Neu-roscan Quik-Cap, which has 40 channels. A Neuroscan NuAmps device was used to collect EEG data at a sampling rate of 1000 Hz. Initially, the electrodes were grounded to the vertex and referenced to the right mastoid (A2 channel).

### 2.2. Data Processing

The amplitude of the high-frequency band increases and the amplitude of the low-frequency band decreases. EEG signals reflect the activity state of the brain. The state of the oceanaut’s mental burden may be indicated by variations in the amplitude of each frequency band of EEG activity. 

**Figure 1:**
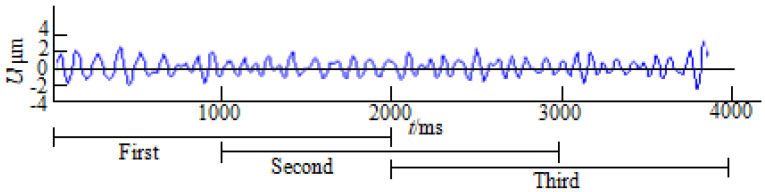
Segmentation of EEG signals.

EEG Signal Processing: The EEG signals were filtered using a 0.5–35 Hz bandpass digital filter, and the data were segmented into 2 s epochs with 50% overlap (1024 points per epoch). The ocular artifact rejection method [27] was used to automatically eliminate the eye blink artifacts, and the remaining epochs were manually rejected (see Figure 1).

(1) $$f[m]=\left\{\begin{array}{l} \sum_{n=0}^{N=1}f[n]W_{N}^{kn},\;0\leq m\leq N-1\\ 0,\;\mathrm{otherwise} \end{array}\right.$$

where $W_{N}=\cos\left(2\pi /N\right)-j\sin\left(2\pi /N\right)$, and *n* is the sample size of EEG signal. 

Delta (0.5–4 Hz), Theta (4–7 Hz), Alpha (7–13 Hz), and Beta (13–30 Hz) are the four types of wave amplitude sequences extracted from f[k]. The amplitude of the four bands in one time window was obtained; this average value was then used as the representative value of the frequency band across fifty-nine time windows. Similarly, the EEG signals of the q electrodes were all processed using the abovementioned method; then, the corresponding 4×q EEG parameters were obtained, denoted as $x_{j}\;\left(1\leq j\leq 4\times q\right)$.

## 3. Methodology

### 3.1. Support Vector Machine

**Figure 2:**
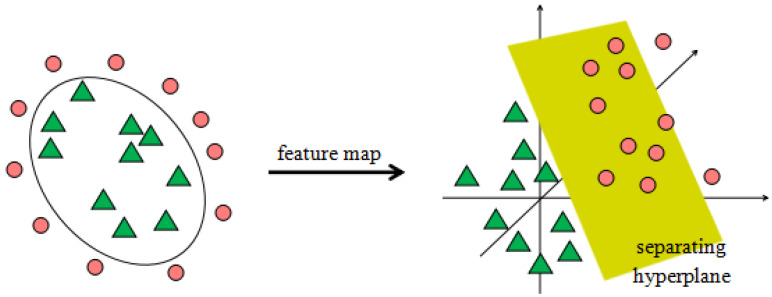
The mapping of feature vectors from a low dimension to a higher dimension.

An SVM training method [28,29,30] creates a model that categorizes fresh instances according to one of two categories given a series of training examples that have each been tagged as belonging to one of the categories. This makes the algorithm a non-probabilistic binary linear classifier. An SVM model is a mapping of the instances as points in space with as much space between the examples of the various categories as feasible. Then, depending on which side of the gap they fall, new samples are projected into that same area and are predicted to belong to a category [31,32,33]. A linearly separable problem is shown in Figure 2. 

Given sample set $A=\left\{x_{i},y_{i}\left|i=1,\cdots ,n\right.\right\}$, where $x_{i}∊R^{n}$ is the input vector, $y_{i}∊\left\{+1,-1\right\}$, m is the sample number.

The input data are mapped from the original space to high-dimensional feature space via the nonlinear mapping function, and the optimal classification hyperplane is constructed in high-dimensional feature space.

(2) $$f\left(x\right)=w\phi \left(x\right)+b=\sum_{k=1}^{n}w_{k}\phi \left(x_{k}\right)+b=0$$

where w is the normal vector to the hyperplane and b is the offset vector of the classification hyperplane. To ensure the accuracy of the classification, the slack variable is introduced. Then, the optimization problem can be expressed as:

(3) $$\left\{\begin{array}{l} min\frac{1}{2}{\left\|w\right\|}^{2}+C\sum_{i=1}^{n}\varepsilon_{i}\;(\varepsilon_{i}\geq 0)\\ s.t.\left\{\begin{array}{l} y_{i}(wx_{i}+b)\geq 1-\varepsilon_{i}\\ C\geq 0 \end{array}\right.(i=1,2,\cdot \cdot \cdot ,n) \end{array}\right.$$

where ε is the slack variable and *C* is the penalty factor.

Introducing kernel function $k(x_{i},x_{j})$, $k(x_{i},x_{j})$ is satisfied with Mercer conditions, and the original problem of solving the optimal hyperplane is transformed into solving the quadratic optimization problem.

(4) $$\max Q(a)=\sum_{i=1}^{n}a_{i}-\frac{1}{2}\sum_{i,j=1}^{n}a_{i}a_{j}y_{i}y_{j}k(x_{i}x_{j})$$

The constraint condition is:

(5) $$\sum_{i=1}^{n}a_{i}y_{i}=0,\;0\leq a_{i}\leq C;i=1,2,\cdots ,n$$

Then, the decision function can be written as:

(6) $$f(x)=sign(\sum_{i,j=1}^{n}a_{i}y_{i}k(x_{i},y_{j})+b)$$

(7) $$K(x_{i},x_{j})=\exp(\frac{-{\left\|x_{i}-x_{j}\right\|}^{2}}{2\sigma^{2}})=\exp(\frac{-{\left\|x_{i}-x_{j}\right\|}^{2}}{g})$$

### 3.2. Multiclass Support Vector Machines

**Figure 3:**
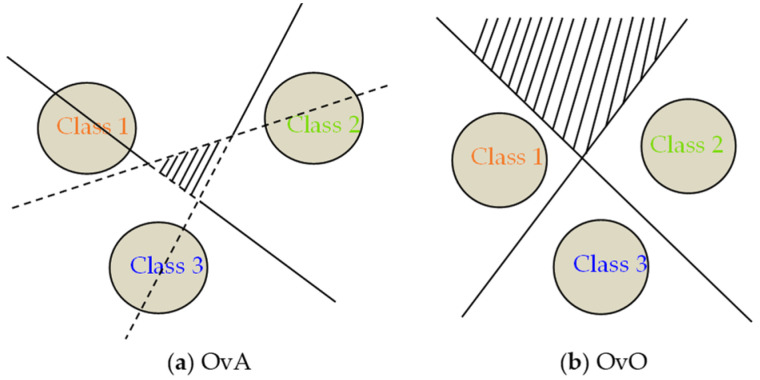
The possible ambiguity regions with (**a**) OvA and (**b**) OvO.

The SVM was initially intended to be a binary classifier. In the literature, one-versus-one (OvO) and one-versus-all (OvA) are the most popular multiclass approaches [34]. By building N binary classifiers using the OvA technique, each classifier is trained to differentiate between two of the N possible classes. In this method, a single classifier is trained for each class, with the samples belonging to that class serving as positive samples and the others serving as negative samples. The unknown sample is then categorized as the sample with the highest classification function value. For an N-way multiclass issue, the OvO builds binary classifiers that are *N(N-1)/2* in size. Each classifier must learn to differentiate between the positive and negative classifications after being trained on two classes of data. At prediction time, a voting procedure is used: all *N(N-1)/2* classifiers are applied to an unseen sample, and the combined classifier predicts the class that received the most “+1” predictions. Graphical representations of OvA binarization and OvO binarization are shown in Figure 3a,b, respectively. Typically, OvO outperforms OvA in terms of categorization accuracy. OvO is utilized in this context to identify mental strain.

### 3.3. Kernel Function Parameter Optimization Based on Quantum Genetic Algorithm

#### 3.3.1. Procedure Description

Although the final classification accuracy is significantly influenced by the choice of kernel function parameter, no comprehensive theory to address the issue has yet been developed. The algorithm of swarm intelligent optimization is used to choose kernel parameters. The best kernel function parameters, like GA and PSO, are adaptively chosen by the swarm intelligence algorithm by using its superior optimization capabilities. Although PSO also has certain drawbacks, such as weak local optimization capabilities, GA has the problems of sluggish convergence and easy local optimum trapping [32,33]. A novel method called QGA combines classical GA with the probability and workings of quantum computing. Chromosome representation in QGA is carried out using quantum bit coding, and the evolutionary search is finished using the quantum gate function and updates. Fast convergence speed, powerful global optimization, and a small population size without hurting algorithm performance are some of its characteristics. As a result, the QGA technique is utilized to adaptively choose the best kernel parameters [35,36]. The specific steps of kernel parameter optimization are as follows: 

The kernel parameters are seen as a chromosome by QGA in step 1. The first population is created by generating N chromosomes at random and encoding the kernel parameters using quantum bits.

(8) $$Q\left(t\right)=\left(q_{1}^{t},q_{2}^{t},\cdots ,q_{N}^{t}\right)$$

(9) $$q_{i}^{t}=\left[\begin{array}{cccc} \alpha_{1}^{t} & \alpha_{2}^{t} & \cdots & \alpha_{m}^{t}\\ \beta_{1}^{t} & \beta_{2}^{t} & \cdots & \beta_{m}^{t} \end{array}\right]$$

where $q_{i}^{t}$ represents the *i* individual of the population that evolved to the t generation. 

One of the quantum bits is represented as:$\left|\varphi \right\rangle =\alpha \left|0\right\rangle +\beta \left|1\right\rangle$, satisfying the normalization condition:

$${\left|\alpha \right|}^{2}+{\left|\beta \right|}^{2}=1$$

Step 2 entails measuring each member of the original population *Q(t)* in binary and transforming it into a population *P(t)* made up of binary strings of length m. 

Step 3 is to evaluate the population *P(t)*’s fitness. The kernel function is changed to accommodate each kernel parameter value, and the MSVM is then used to categorize the tested data set and assess the fitness. The values of the kernel parameters and the corresponding fitness under the current optimal fitness (optimum classification accuracy) are kept, i.e., the current ideal person. 

Step 4. *Q(t)* is updated to create a fresh population using the quantum gate operation. Choosing a quantum rotating gate directs the program to look in the right place for the best answer. The quantum rotating gate’s updating procedure is as follows:

(10) $$\left[\begin{array}{c} \alpha_{i}^{t+1}\\ \beta_{i}^{t+1} \end{array}\right]=U\left(\theta_{i}\right)\left[\begin{array}{c} \alpha_{i}^{t}\\ \beta_{i}^{t} \end{array}\right]=\left[\begin{array}{cc} \cos\theta_{i} & -\sin\theta_{i}\\ \sin\theta_{i} & \cos\theta_{i} \end{array}\right]\left[\begin{array}{c} \alpha_{i}^{t}\\ \beta_{i}^{t} \end{array}\right]=\left[\begin{array}{c} \alpha_{i}^{t}\cos\theta_{i}-\beta_{i}^{t}\sin\theta_{i}\\ \alpha_{i}^{t}\sin\theta_{i}+\beta_{i}^{t}\cos\theta_{i} \end{array}\right]$$

$U\left(\theta_{i}\right)$ is the quantum revolving gate; it is expressed as follows:

(11) $$U\left(\theta_{i}\right)=\left[\begin{array}{cc} \cos\theta_{i} & -\sin\theta_{i}\\ \sin\theta_{i} & \cos\theta_{i} \end{array}\right]$$

θ is the rotation angle; its size and direction are determined by the rotation angle adjustment strategy.

Step 5. Let the evolution algebra be *t* = *t* + 1 and return to step 3 to continue.

Step 6. Output the optimal parameters and test the test data with the optimal parameters.

#### 3.3.2. Key Parameter Setting

Some important algorithmic parameters, such as the following ones, need to be initialized before utilizing QGA to achieve the best MSVM kernel function settings. For the initialization population, each $\left(\alpha_{i}^{t},\beta_{i}^{t}\right)$ in Q(t) is initialized to $\left(1/\sqrt{2},1/\sqrt{2}\right)$, and all states of a chromosome are superimposed with the same probability at the time of initial evolution.

For the fitness function, the purpose of using QGA to optimize MSVM is to obtain the optimal classification accuracy. Therefore, the accuracy of classification is selected as the fitness function in the parameters of the QGA optimization kernel function.

(12) $$\mathrm{Fitness}=\mathrm{Acc}\left(\alpha_{i}^{t},\beta_{i}^{t}\right)$$

where $\mathrm{Acc}(\alpha_{i}^{t},\beta_{i}^{t})$ is the final classification accuracy of MSVM.

For the quantum spinning gate’s angle, a general adjustment method is used to alter the value of the rotation angle since it impacts how quickly the optimization algorithm converges [37,38,39]. The data table of rotation angle adjustment is shown in Table 1. For the algorithm’s final condition, the optimization procedure for the kernel parameter ends when the maximum number of iterations exceeds the original set algebra or when the absolute value of the difference between the best fitness (classification accuracy) for 10 successive generations is less than 0.001.

#### 3.3.3. Simulation Analysis

Four standard data sets from the UCI standard database—wine, iris, appendicitis, and glass—were chosen as experimental data in order to assess the efficacy of QGA in the optimization of MSVM kernel parameters. The experimental data were categorized using QGA, GA, and PSO, respectively. The quantity of samples, dimensions, and categories for the four-test data in the UCI data sets are shown in Table 2.

**Figure 4:**
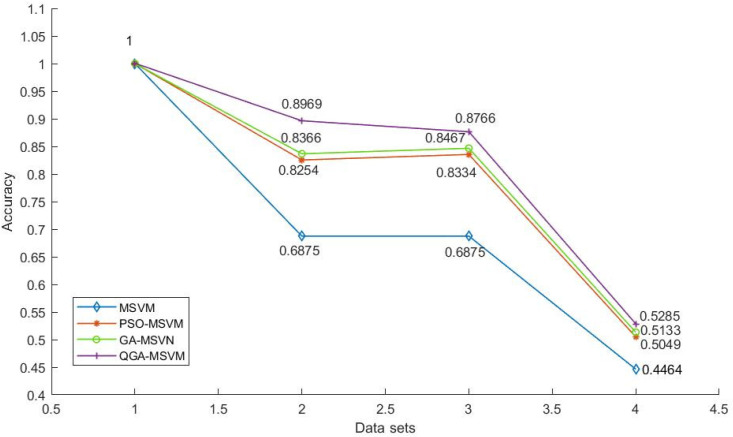
The accuracy of four algorithms.

The average classification accuracy after 50 classification simulations of four test data from the UCI data sets is shown in Figure 4. Figure 4 shows the accuracy of the MSVM classification optimized by the three distinct methods for the four test data sets of wine, iris, appendicitis, and glass. While GA-MSVM and PSO-MSVM have varying test results for various types of data, QGA-MSVM has the greatest average classification accuracy. This result showed that, while the GA and PSO algorithms have some issues, such as kernel function parameter values falling into the local optimum, premature convergence, which do not converge to the optimal kernel parameter value, the QGA algorithm can adaptively select the best kernel function parameters each and every time during the testing process.

## 4. Experimental Method

### 4.1. Participants

**Figure 5:**
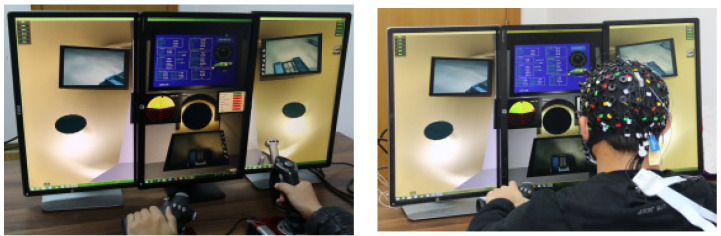
Simulated operation.

Eight healthy people (eight men, mean age 24.8 ± 1.8 years, average height 171.8 ± 6.7 cm, middleweight 651 ± 0.4 kg) took part in our research. All individuals had normal or corrected-to-normal eyesight and were right-handed. None of them had a history of neurological or mental illnesses [40,41]. In the experiment, the Ag-AgCI disc-like electrode was applied using Neuroscan, and the EEG data were recorded using the worldwide standard 10–20 system electrode insertion technique. The sampling rate was 1000 hertz (Hz). The potential change on the scalp during the measurement traveled via the wire from the electrode to the electroencephalograph through the conductive paste. The electrode put on the body, such as the earlobe, served as the reference electrode. The working electrode placed on the scalp served as the working electrode, and the difference value between the working electrode and the reference electrode was the final EEG signal that was recorded (see Figure 5).

### 4.2. Design of the Task

**Figure 6:**
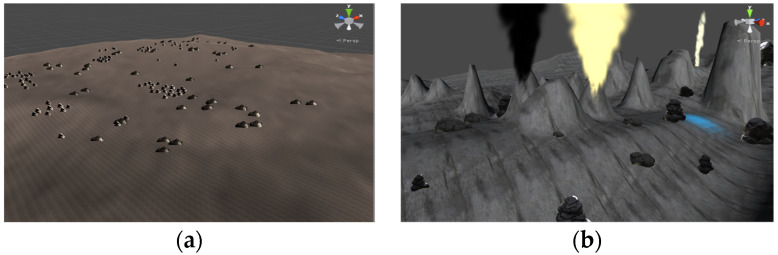
Terrain of sea basin and hydrothermal field. (**a**) Terrain of sea basin; (**b**) terrain of hydrothermal field.

Driving duties were split up into three separate scenarios throughout the experiment: the sea basin region, the seamounts area, and the hydrothermal area. The hydrothermal region requires additional hydrothermal vents and other impediments since the landscape there is hilly with significant changes, unlike the sea basin area, which has a relatively level plain topography (see Figure 6).

**Figure 7:**
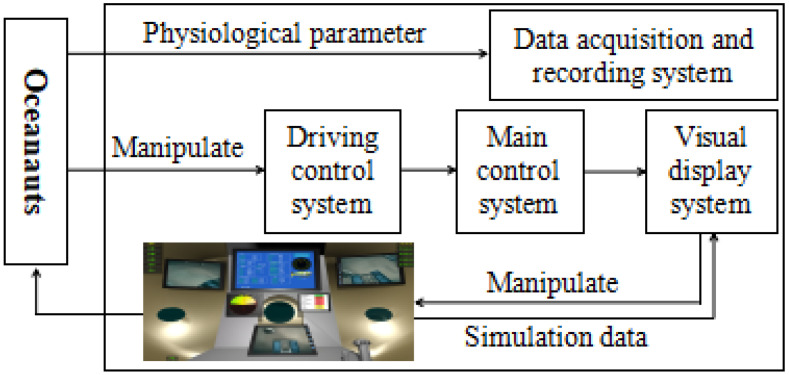
Experiment platform.

Participants had to operate the submersible to avoid obstacles, arrive at the stated location within the allotted time, and attempt to maintain a height above the ground between 2 and 6 m. On the imaging sonar, the target’s position was dynamically presented. The mission was completed when the indicated site was reached or after the allotted three minutes. On the first day, the fifth day, and the tenth day, the same exam was given to each participant three times. Figure 7 depicts the experiment platform for the driving operation task. 

According to subjective assessments of the mental burden, task complexity levels were categorized into three groups. The mental effort levels of the three conditions were created using the three-task experiment. Driving in the sea basin region has a low workload, driving near seamounts takes medium effort, and driving near hydrothermal areas has a high workload.

## 5. Results and Discussion

EEG physiological data were obtained and evaluated for this investigation. Various EEG workload indicators may be used to diagnose various elements of a participant’s cognitive–energetic status. As a result, in order to employ EEG indices for assessing operator state, it is necessary to understand what each indicator means in connection to the relevant task. Before EEG indices may be routinely used for applications, the further investigation of the fluctuation of diagnosticity with task demands is required (see Table 3). 

Physiological index changes were measured before, during, and after the simulated operation.

**Figure 8:**
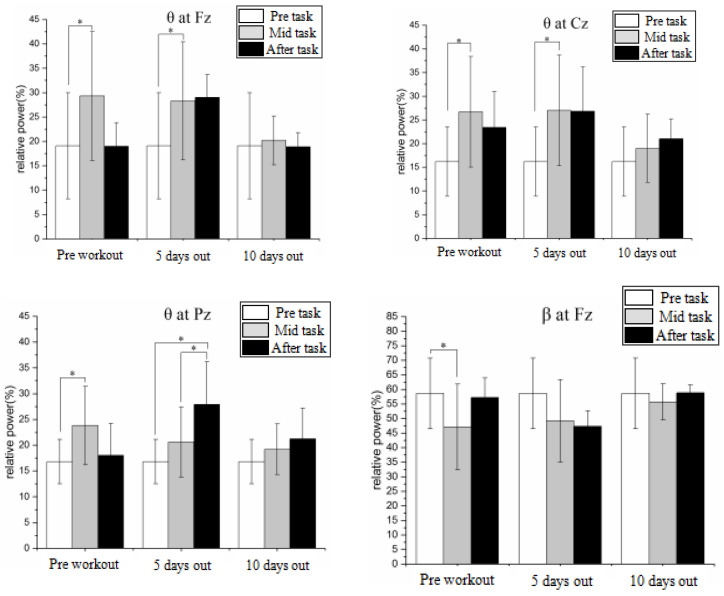

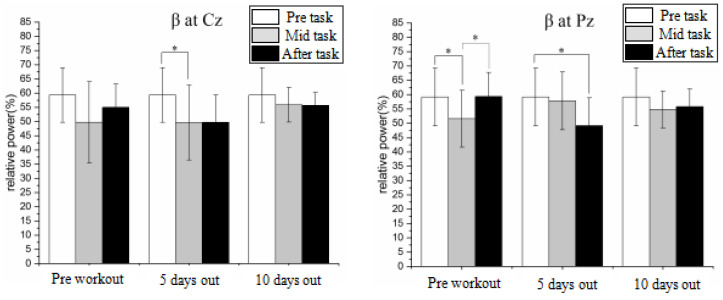
Physiological index changes of simulated tasks. * *p* < 0.05.

There is a statistically significant increase in the relative power of the wave of Fz in the operation compared to both pre- and post-training levels (*p* < 0.05), an increase in the relative power of the wave of Cz and Pz compared to pre-training levels (*p* < 0.05), and a statistically significant decrease in the power of the wave of Fz and Pz compared to pre-training levels (*p* < 0.05). The strength of the wave of Cz is substantially lower than before the surgery after 5 days of training (*p* < 0.05), whereas the waves of Fz and Cz are both significantly greater. After the operation, the relative power of the Pz wave is much greater than it was before, and during the operation (*p* < 0.05), the relative power of the wave is significantly higher than it was before. None of these modifications took place after 10 days of training. With the help of the aforementioned analysis, we could classify the mental burden of oceanauts using the EEG after five days of training. We then chose the training and test data and used KNN, BP, Random Forest, SVM, and QGA-MSVM (see Figure 8).

Table 4 makes clear that there are obvious individual variances among the eight patients. KNN has the greatest classification accuracy (73.6%), BP has the highest classification accuracy (80.2%), RF has the best classification accuracy (75.9%), and SVM has the highest classification accuracy (73.6%). The average classification accuracy for each model is SVM > BP > RF > KNN, with a classification accuracy rate of 79.6%. The SVM classification impact is superior in terms of accuracy rate. Even if the SVM classification model’s classification has improved, the average accuracy still needs to be raised. The penalty parameter C and the kernel width g have a major role in the success of the SVM algorithm. The proper C and g values are difficult to predict in advance. The findings show that the QGA-MSVM model’s recognition accuracy is much better than that of KNN, BP, RF, and SVM; the model’s average accuracy is 91.8%. As a result, the QGA-MSVM model is better suited for identifying mental burden based on the data presented above.

## 6. Conclusions

This research proposes a QGA-based technique for MSVM kernel function parameter optimization. QGA adaptively selects the best kernel parameters because of its great global search capability, variety of population, quick convergence speed, and quick parallel processing time. The simulation results demonstrate that the performance of QGA-MSVM is superior to that of PSO and GA using the four data sets from the UCI standard data set as measured data. Statistics were used to analyze EEG data from driving simulation operating experiments conducted in sea basin, seamount, and hydrothermal areas. After 5 days of training, EEG data were chosen, and QGA-MSVM was used to determine the mental burden of oceanauts. The findings demonstrate that the QGA-MSVM algorithm is capable of accurately detecting the oceanauts’ mental workload while they are operating a vehicle. The average accuracy of the QGA-MSVM model is 91.8%, and it serves as a foundation for an intelligent evaluation of the oceanauts’ mental workload.

In addition to navigating past hazards and risky locations when diving, oceanauts must also use complicated equipment within the manned compartment to carry out duties like setting up markers and collecting sediment and water samples. Oceanauts must also perform diving activities, create and modify dive plans depending on the topography of the seabed, and continuously check system status. 

During deep diving missions lasting more than 10 hours, Oceanauts dive many kilometers down in a cramped and small workspace inside the manned compartment, necessitating extended hours of observation and unbroken operating. They continue to labor in a very stressful condition, mostly carrying cognitive burdens.

Time constraints, safety concerns (complicated deep-sea topography), environmental considerations (abnormal temperature, vibration, acceleration, or noise), and the difficulty of the jobs all affect how much cognitive work they must do. Their productivity is greatly reduced when the cognitive load is too high or too low, which may result in a variety of operational mistakes, neglect, disorder, physio-logical stress, and even serious safety mishaps. 

It is crucial to accurately gauge the cognitive load level of oceanauts in order to minimize human mistakes, ensure diving safety, improve the design of human–machine interfaces, and select and train oceanauts. Additionally, this offers helpful references for the creation of portable oceanauts for the real-time cognitive load monitoring of divers.

Future research will include more thorough physiological data collection from submarine divers during actual diving operations. This study may further our understanding of the physiological, psychological, and cognitive demands placed on submarine divers in real-world operational settings. 

The more precise measurement and assessment of the cognitive load of submarine divers in undersea robotic systems may be aided by the creation of an individual database on submarine divers, which can be established; the Submarine Simulator Operating System’s improvement; the use of mechanical arms and submarine motion equations to more accurately calculate their movements; increasing the visual effects’ realism by further optimizing them both inside and outside the submarine’s cabin; and making the simulation operating system more realistic and immersive thanks to VR design. The following are particular ways to improve the cognitive load recognition model: increasing the amount of data and parameters used to quantify cognitive load and train the recognition model; and increasing the precision of load categorization, considering and enhancing more complicated machine learning techniques. Currently, the tasks in the simulated tests do not completely cover all the execution processes since operating and driving mechanical arms is a difficult undertaking. The cognitive burden of operators will vary depending on the work; hence, more difficult tasks need additional research.

## 7. Contributions

In order to develop a reasonable operation process and to effectively intervene when the mental load of the underwater crew is too small or too large (man–machine function redistribution, the responsibility redistribution of the chief and co-pilot, and task strategy reselection), physiological indicators are used. Preventing occupational illnesses and pilot weariness, safeguarding their physical and mental health, lowering human mistake rates, and enhancing the safety of underwater navigation are all very important aspects.

## Figures and Tables

**Table 1:** Rotation angle adjustment strategy.

$x_{j}$	$best_{j}$	$f\left(x\right)\;>\;f\left(best\right)$	$\Delta \theta_{j}$	$s\left(\alpha_{j},\beta_{j}\right)$			
				$a_{j}\beta_{j}\;>\;0$	$a_{j}\beta_{j}\;<\;0$	$a_{j}\;=\;0$	$\beta_{j}\;=\;0$
0	0	FALSE	0	0	0	0	0
0	0	TRUE	0	0	0	0	0
0	1	FALSE	0.01π	+1	−1	0	±1
0	1	TRUE	0.01π	−1	+1	±1	0
1	0	FALSE	0.01π	−1	+1	±1	0
1	0	TRUE	0.01π	+1	−1	0	±1
1	1	FALSE	0	0	0	0	0
1	1	TRUE	0	0	0	0	0

**Table 2:** The UCI standard data sets for test.

Data Set	Number of Samples	The Sample Dimensions	Number of Categories
Wine	178	13	3
Iris	150	4	3
Appendicitis	106	7	2
Glass	214	9	6

**Table 3:** EEG changes before, during, and after simulated operation.

Frequency Band	Position	Pre Workout	5 Days Out	10 Days Out
θ	Fz	29.3 ± 13.2	28.3 ± 12.1	20.2 ± 5.0 *^#^
	Cz	26.5 ± 11.7	27.0 ± 11.7	19.0 ± 7.2 *^#^
	Pz	23.8 ± 4.4	20.62 ± 6.8	19.2 ± 4.9 *
β	Fz	47.1 ± 14.7	49.2 ± 14.2	55.7 ± 6.2 *^#^
	Cz	49.7 ± 14.3	49.1 ± 13.3	55.9 ± 6.1 *^#^
	Pz	51.5 ± 9.9	57.8 ± 10.1 *	54.7 ± 6.4

Compared to before training * *p* < 0.05; compared to five days after training ^#^ *p* < 0.05.

**Table 4:** Results based on EEG signals.

Participants	Driving Task				
	KNN	BP	Random Forest	SVM	QGA-MSVM
01	70.5%	79.2%	78.5%	78.7%	92.1%
02	72.9%	79.0%	69.6%	77.2%	92.3%
03	67.5%	69.2%	77.4%	79.4%	83.8%
04	80.1%	83.6%	82.4%	86.1%	86.5%
05	62.6%	77.8%	67.3%	81.8%	92.1%
06	72.7%	83.5%	80.8%	77.4%	84.9%
07	82.3%	89.3%	78.2%	79.2%	85.3%
08	79.8%	75.5%	72.7%	81.5%	80.5%
Mean	73.6%	79.6%	75.9%	80.2%	91.8%

## Data Availability

The data that support the findings of this study are available on request fromthe corresponding author The data arenot publicly available due to privacy orethical restrictions.
